# Design and Evaluation of Fast Dissolving Tablets of Clonazepam

**DOI:** 10.4103/0250-474X.49125

**Published:** 2008

**Authors:** S. B. Shirsand, Sarasija Suresh, P. V. Swamy, D. Nagendra Kumar, M. V. Rampure

**Affiliations:** Department of Pharmaceutical Technology, H. K. E. Society's College of Pharmacy, Sedam Road, Gulbarga-585 105, India; 1 Department of Pharmaceutics, Al-Ameen College of Pharmacy, Near Lalbagh Main Gate, Hosur Road, Bangalore-560 027, India; 2 S.V.E.T.'s College of Pharmacy, Humnabad-585 330, India

**Keywords:** Fast dissolving tablets, clonazepam, crospovidone, croscarmellose sodium, sodium starch glycolate

## Abstract

In the present work, fast dissolving tablets of clonazepam were prepared by direct compression method with a view to enhance patient compliance. Three super-disintegrants, viz., crospovidone, croscarmellose sodium and sodium starch glycolate in different ratios with microcrystalline cellulose (Avicel PH-102) along with directly compressible mannitol (Pearlitol SD 200) to enhance mouth feel. The prepared batches of tablets were evaluated for hardness, friability, drug content uniformity, wetting time, water absorption ratio and *in vitro* dispersion time. Based on *in vitro* dispersion time (approximately 13 s), three formulations were tested for the *in vitro* drug release pattern (in pH 6.8 phosphate buffer), short-term stability (at 40°/75% relative humidity for 6 mo) and drug-excipient interaction (IR spectroscopy). Among the three promising formulations, the formulation prepared by using 10% w/w of crospovidone and 35% w/w of microcrystalline cellulose emerged as the overall best formulation (t_50%_ 1.8 min) based on the *in vitro* drug release characteristics compared to conventional commercial tablet formulation (t_50%_ 16.4 min). Short-term stability studies on the formulations indicated that there were no significant changes in drug content and *in vitro* dispersion time (*P*<0.05).

Many patients express difficulty in swallowing tablets and hard gelatin capsules, resulting in non-compliance and ineffective therapy^1^. Recent advances in novel drug delivery systems (NDDS) aim to enhance safety and efficacy of drug molecules by formulating a convenient dosage form for administration and to achieve better patient compliance. One such approach led to development of fast dissolving tablets^2^–^4^. Advantages of this drug delivery system include administration without water, convenience of administration and accurate dosing as compared to liquids, easy portability, ability to provide advantages of liquid medication in the form of solid preparation, ideal for pediatric and geriatric patients and rapid dissolution/absorption of the drug, which may produce rapid onset of action. Some drugs are absorbed from mouth, pharynx and esophagus as the saliva passes down into the stomach and in such cases bioavailability of the drug is increased: pre-gastric absorption can result in improved bioavailability and as result of reduced dosage, improved clinical performance through a reduction of unwanted effects. Clonazepam is a benzodiazepine derivative with marked antiepileptic properties. It may be used in the treatment of all types of epilepsy and seizures. It is also indicated in mycoclonus and associated abnormal movements, and for the treatment of panic disorders^5^. It was selected as drug candidate, since it is not available in such dosage form. Aim of the present study was to develop fast dissolving tablets of clonazepam by simple and cost effective direct compression technique.

Clonazepam (CZ) and the super-disintegrants (crospovidone, croscarmellose sodium and sodium starch glycolate) were the gift samples from Torrent Pharma, Ahmedabad and Wockhardt Research Centre, Aurangabad respectively. Directly compressible mannitol (Pearlitol SD 200), microcrystallinecellulose (MCC) and sodium stearylfumarate (SSF) were generous gifts from Strides Acrolabs, Bangalore. All other chemicals used were of Analytical Reagent grade.

Fast dissolving tablets of CZ were prepared by direct compression^6^ according to the formulae given in Table 1. All the ingredients were passed through # 60 mesh separately. Then the ingredients were weighed and mixed in geometrical order and compressed into tablets of 150 mg using 8 mm round flat punches on 10-station rotary tablet machine (Clit, Ahmedabad). A batch of 60 tablets was prepared for each of the designed formulations.

**TABLE 1:** COMPOSITION OF DIFFERENT BATCHES OF FAST DISSOLVING TABLETS OF CLONAZEPAM

Formulation	Ingredients (mg)*								
	
code	CZ	Cros-povidone	Cros-carmellose sodium	Sodium starch glycolate	MCC	SSF	Talc	Aspart ame	Pearlitol SD200
DC_0_	2	--	--	--	--	1.5	3	3	140.5
DCP_1_	2	3	--	--	--	1.5	3	3	137.5
DCP_2_	2	6	--	--	30.0	1.5	3	3	104.5
DCP_3_	2	9	--	--	37.5	1.5	3	3	94.0
DCP_4_	2	12	--	--	45.0	1.5	3	3	83.5
DCP_5_	2	15	--	--	52.5	1.5	3	3	73.0
DCCS_1_	2	--	3	--	--	1.5	3	3	137.5
DCCS_2_	2	--	6	--	30.0	1.5	3	3	104.5
DCCS_3_	2	--	9	--	37.5	1.5	3	3	94.0
DCCS_4_	2	--	12	--	45.0	1.5	3	3	83.5
DCCS_5_	2	--	15	--	52.5	1.5	3	3	73.0
DSSG_1_	2	--	--	3	--	1.5	3	3	137.5
DSSG_2_	2	--	--	6	30.0	1.5	3	3	104.5
DSSG_3_	2	--	--	9	37.5	1.5	3	3	94.0
DSSG_4_	2	--	--	12	45.0	1.5	3	3	83.5
DSSG_5_	2	--	--	15	52.5	1.5	3	3	73.0

* All the quantities expressed are in mg/tablet. CZ is clonazepan, MCC represents microcrystalline cellulose, SSF is sodium stearylfumarate, Pearlitol SD200 is the directly compressible mannitol. Formulations DCP_5_, DCCS_5_ and DSSG_5_ were selected as the promising and used for further studies.

For the weight variation twenty tablets were selected at random and assessed individually. The individual weights were compared with the average weight for determination of weight variation^7^. Hardness and friability of the tablets were determined by using Monsanto hardness tester and Roche friabilator, respectively. For content uniformity test, ten tablets were weighed and powdered. The powder equivalent to 2 mg of clonazepam was extracted into methanol and liquid was filtered (Whatmann No. 1 filter paper). The CZ content in the filtrate was determined by measuring the absorbance at 308 nm after appropriate dilution with methanol. The drug content was determined using the standard calibration curve. The mean percent drug content was calculated as an average of three determinations^8^. For determination of wetting time and water absorption ratio^9^, a piece of tissue paper folded twice was placed in a small petridish (internal diameter of 5 cm) containing 6 ml of water. A tablet was placed on the paper and the time required for complete wetting was measured. The wetted tablet was then weighed. Water absorption ratio ‘R’ was calculated using the eqn. R=100×(W_b_–W_a_)/W_a_, where W_a_ is weight of tablet before water absorption and W_b_ is weight of tablet after water absorption. For determination of *in vitro* dispersion time, one tablet was placed in a beaker containing 10 ml of pH 6.8 phosphate buffer at 37±0.5° and the time required for complete dispersion (with mild shaking) was determined^10^. IR spectra of CZ and its formulations were obtained by potassium bromide pellet method using Perkin-Elmer FTIR series (Model 1615) spectrophotometer in order to rule out drug-carrier interactions.

*In vitro* dissolution of CZ fast dissolving formulated tablets and one commercial conventional tablet was studied in USP XXIII type-2 dissolution apparatus (Electrolab, Model-TDT 06N) employing a paddle stirrer at 50 rpm using 900 ml of pH 6.8 phosphate buffer at 37±0.5° as dissolution medium^11^. One tablet was used in each test. Aliquots of dissolution medium (5 ml) were withdrawn at specific intervals of time and analyzed for drug content by measuring the absorbance at 307.5 nm. The volume withdrawn at each time interval was replaced with fresh quantity of the dissolution medium. Cumulative percent of CZ released was calculated and plotted against time.

Short-term stability studies on the promising formulations (DCP_5_, DCCS_5_ and DSSG_5_) were carried out by storing the tablets at 40°/75% RH over a 6 mo period according to ICH guidelines. At intervals of 1, 3 and 6 mo, the tablets were visually examined for any physical changes, changes in drug content and *in vitro* dispersion time.

Fast dissolving tablets of CZ were prepared by direct compression method employing crospovidone, croscarmellose sodium and sodium starch glycolate as super-disintegrants in different ratios with microcrystalline cellulose. Directly compressible mannitol (Pearlitol SD 200) was used as a diluent to enhance mouth feel. A total of fifteen formulations and a control formulation DC_0_ (without super-disintegrant) were designed. As the blends were free flowing (angle of repose <30°, and Carr's index <15%) tablets obtained were of uniform weight (due to uniform die fill), with acceptable variation as per IP specification i.e., below 7.5% (Table 2). Drug content was found to be in the range of 96 to 102%, which is within acceptable limits. Hardness of the tablets was found to be 2.6 to 3.5 kg/cm². Friability below 1% was an indication of good mechanical resistance of the tablets. Water absorption ratio and wetting time, which are important criteria for understanding the capacity of disintegrants to swell in the presence of little amount of water were found to be in the range of 64-84% and 22-94 s, respectively. Among all the designed formulations, three formulations, viz., DCP_5_, DCCS_5_ and DSSG_5_ were found to be promising and showed *in vitro* dispersion time ranging from 13 to 20 s, which facilitated their faster dispersion in the mouth.

**TABLE 2:** EVALUATION OF FAST DISSOLVING TABLETS

Formulation	Test						
	
code	Hardness* (kg/cm^2^)	Friability (%)	Thickness (mm)	*In vitro* dispersion time* (s)	Wetting time* (s)	Water absorption ratio* (%)	Percent drug content*
DC_0_	3.5±0.244	0.54	2.40	2.54±3.0	255.0±1.58	49.28±4.66	97.02±0.81
DCP_1_	3.3±0.288	0.74	2.48	86.0±3.50	94.0±2.0	64.93±1.51	99.18±0.62
DCP_2_	3.2±0.258	0.80	2.60	40.0±1.52	41.59±1.64	75.77±2.79	100.53±2.00
DCP_3_	3.2±0.256	0.73	2.50	26.0±1.52	27.0±1.12	79.87±2.25	101.14±0.91
DCP_4_	3.3±0.215	0.66	2.62	18.0±1.0	22.0±1.23	82.06±0.77	99.25±0.53
DCP_5_	3.0±0.286	0.57	2.48	13.0±1.0	14.88±0.83	84.13±2.42	97.02±0.81
DCCS_1_	2.9±0.10	0.58	2.25	48.0±3.21	58.0±2.0	74.92±1.22	97.35±2.64
DCCS_2_	2.8±0.90	0.63	2.35	35.0±1.0	42.0±3.05	79.55±3.59	100.53±2.00
DCCS_3_	2.9±0.13	0.65	2.50	25.0±1.0	30.6±2.08	79.87±2.25	98.91±1.42
DCCS_4_	3.1±0.10	0.60	2.30	20.0±1.52	24.0±1.0	84.13±2.42	102.56±0.84
DCCS_5_	3.0±0.15	0.56	2.50	17.0±1.0	19.66±2.08	82.06±0.77	97.69±1.42
DSSG_1_	3.0±0.20	0.77	2.50	93.3±7.63	94.0±3.50	65.0±2.25	101.01±1.42
DSSG_2_	2.9±0.11	0.73	2.40	49.6±2.08	58.0±2.00	70.0±2.00	96.34±1.42
DSSG_3_	3.0±0.20	0.67	2.62	41.60±2.08	43.0±1.50	75.0±2.0	97.42±1.06
DSSG_4_	2.76±0.25	0.70	2.48	32.0±1.52	34.0±3.0	79.0±1.51	98.09±0.62
DSSG_5_	2.6±0.17	0.64	2.52	20.0±2.10	22.0±1.00	82.0±2.42	101.34±2.02

* Average of three determinations

Overall, the formulation DCP_5_ containing 10% w/w of crospovidone and 35% w/w of microcrystalline cellulose was found to be promising and has shown an *in vitro* dispersion time of 13 s wetting time of 15 s and water absorption ratio of 84% when compared to control formulation (DC_0_) which showed 254 s, 255 s and 49% respectively for the above parameters (Table 2).

*In vitro* dissolution studies on the promising formulations (DCP_5_, DCCS_5_ and DSSG_5_), the control (DC_0_) and commercial conventional formulations (CCF) were carried out in pH 6.8 phosphate buffer, and the various dissolution parameter values viz., percent drug dissolved in 5 min, 10 min and 15 min (D_5_, D_10_ and D_15_), dissolution efficiency at 10 min (DE_10 min_)^12^, t_50%_, t_70%_ and t_90%_ are shown in Table 3 and the dissolution profiles depicted in fig. 1. This data reveals that overall, the formulation DCP_5_ has shown ten-fold faster drug release (t_50%_ 1.8 min) when compared to the commercial conventional tablet formulation of CZ (t_50%_ 16.4 min) and released 5-times more drug than the control formulation in 10 min.

**TABLE 3:** *IN VITRO* DISSOLUTION PARAMETERS IN PH 6.8 PHOSPHATE BUFFER

Formulation	Dissolution Parameters						
	
code	D_5_ (%)	D_10_ (%)	D_15_ (%)	DE_10min_ (%)	t_50%_ (min)	t_70%_ (min)	t_90%_ (min)
DC_0_	6	15	17	20.41	> 30	> 30	> 30
DCP_5_	64	78.5	83.5	67.42	1.8	6.8	19.8
DCCS_5_	57.5	67.5	75	54.93	4.2	11.8	28
DSSG_5_	55	68	78.5	51.77	4.8	11	26.2
CCF	29.5	42.5	48.5	33.41	16.4	> 30	> 30

DC_0_=control formulation, CCF=commercial conventional formulation, D_5_=percent drug released in 5 min, D_10_=percent drug released in 10 min, D_15_=percent drug released in 15 min, DE_10min_=dissolution effciency in 10 min, t_50%_=time for 50% drug dissolution, t_70%_=time for 70% drug dissolution, t90%=time for 90% drug dissolution.

**Fig. 1:**
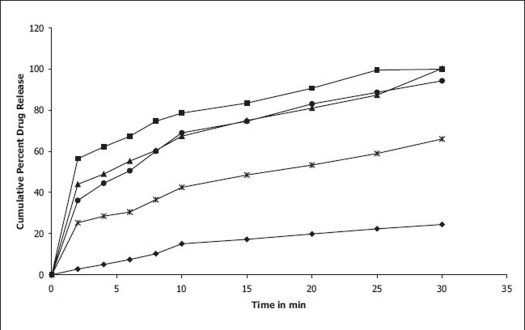
*In vitro* cumulative percent drug release from promising clonazepam formulations *In vitro* cumulative percent drug release Vs time profiles of promising clonazepam  formulations  DC_0_ (–●–),  DCP_5_ (–■–), DCCS_5_ (–▲–), DSSG_5_ (–●–) and CCF (–*–) in pH 6.8 phosphate buffer

IR spectroscopic studies indicated that the drug is compatible with all the excipients. The IR spectrum of DCP_5_ showed all the characteristic peaks of clonazepam pure drug, thus confirming that no interaction of drug occurred with the components of the formulation. Short-term stability studies of the above formulations indicated that there were no significant changes in drug content and *in vitro* dispersion time at the end of 6 mo period (p<0.05).

## References

[1] Seager H (1998). Drug delivery products and the Zydis fast dissolving dosage forms. J Pharm Pharmacol.

[2] Chang RK, Guo X, Burnside BA, Cough RA (2000). Fast dissolving tablets. Pharm Tech.

[3] Dobetti L (2001). Fast-melting tablets: Developments and technologies. Pharma Tech.

[4] Kuchekar BS, Arumugam V (2001). Fast dissolving tablets. Indian J Pharm Educ.

[5] Sweetman SC (2002). Martindale: The complete drug reference.

[6] Kuchekar BS, Badhan AC, Mahajan HS (2004). Mouth dissolving tablets of salbutamol sulphate: A novel drug delivery system. Indian Drugs.

[7] Banker GS, Anderson NR, Lachman L, Lieberman HA, Kanig JL (1987). Tablets. The theory and practice of industrial pharmacy.

[8] (1996). Indian Pharmacopoeia.

[9] Chaudhari PD, Chaudhari SP, Kohle SR, Dave KV, More DM (2005). Formulation and evaluation of fast dissolving tablets of famotidine. Indian Drugs.

[10] Bi YX, Sunada H, Yonezawa Y, Danjo K (1999). Evaluation of rapidly disintegrating tablets by direct compression method. Drug Develop Ind Pharm.

[11] Bhagwati ST, Hiremath SN, Sreenivas SA (2005). Comparative evaluation of disintegrants by formulating cefixime dispersible tablets. Indian J Pharm Edu Res.

[12] Khan KA (1975). The concept of dissolution efficiency. J Pharm Pharmacol.

